# A Spiroligomer α-Helix Mimic That Binds HDM2, Penetrates Human Cells and Stabilizes HDM2 in Cell Culture

**DOI:** 10.1371/journal.pone.0045948

**Published:** 2012-10-18

**Authors:** Zachary Z. Brown, Kavitha Akula, Alla Arzumanyan, Jennifer Alleva, Marcus Jackson, Eugeney Bichenkov, Joel B. Sheffield, Mark A. Feitelson, Christian E. Schafmeister

**Affiliations:** 1 Department of Chemistry, Princeton University, Princeton, New Jersey, United States of America; 2 Chemistry Department, Temple University, Philadelphia, Pennsylvania, United States of America; 3 Department of Biology, Temple University, Philadelphia, Pennsylvania, United States of America; Consiglio Nazionale delle Ricerche, Italy

## Abstract

We demonstrate functionalized spiroligomers that mimic the HDM2-bound conformation of the p53 activation domain. Spiroligomers are stereochemically defined, functionalized, spirocyclic monomers coupled through pairs of amide bonds to create spiro-ladder oligomers [1]. Two series of spiroligomers were synthesized, one of structural analogs and one of stereochemical analogs, from which we identified compound **1**, that binds HDM2 with a Kd value of 400 nM. The spiroligomer **1** penetrates human liver cancer cells through passive diffusion and in a dose-dependent and time-dependent manner *increases* the levels of HDM2 more than 30-fold in Huh7 cells in which the p53/HDM2 negative feed-back loop is inoperative. This is a biological effect that is not seen with the HDM2 ligand nutlin-3a. We propose that compound **1** modulates the levels of HDM2 by stabilizing it to proteolysis, allowing it to accumulate in the absence of a p53/HDM2 feedback loop.

## Introduction

The need for new chemical agents that bind proteins and modulate protein function *in vivo* provides a fundamental challenge for chemists. Molecules that are intermediate in size between small molecule drugs (<600 Daltons) and antibodies (∼150,000 Daltons) could provide new chemical entities capable of binding to the large, flat protein surfaces in a way that could disrupt protein-protein interactions and modulate protein function [2]. α-Helices are the most commonly observed secondary structure in proteins and frequently act as interfacing domains in protein-protein interactions [3], [4]. Synthetic chemists have exerted a great deal of effort to design synthetic α-helix mimics αs potential therapeutic agents and/or biological probes. Strategies for helix mimicry include covalently bonded peptide helices [5], [6], [7], [8], helical β-peptides [9], [10], and nonpeptidic small molecules [11], [12], [13], [14]. Towards this goal, we have developed “spiroligomers” as shape-persistent and shape-programmable scaffolds, which can project functional groups in defined three-dimensional constellations and are synthesized in a convergent fashion [1], [15], [16]. Spiroligomers are highly preorganized macromolecules (600 to 2,000 Daltons), which can be designed to recapitulate the presentation of the relevant side chains of one partner of a protein-protein interaction, bind the other partner and mediate a biological response.

The protein HDM2 is a human E3 ubiquitin ligase and a negative regulator of p53, a protein that is central to the apoptotic pathway and performs a vital role of tumor suppressor through the maintenance of genetic stability [17]. HDM2 binds the N-terminal transactivation domain of p53 and lowers the levels of p53 by ubiquitinating p53, targeting it for degradation via the proteasome. Upon the detection of DNA damage or other cytotoxic stresses, p53 migrates to the nucleus, and initiates either DNA repair or apoptosis. The p53/HDM2 interaction is mediated by the N-terminal helical domain of p53 presenting three hydrophobic residues (Phe19, Trp23 and Leu26) that are buried within a hydrophobic cleft of HDM2 [3]. HDM2 has been extensively investigated as a therapeutic target by both small molecules including nutlin-3a [18] and MI-219 [19] as well as peptides [20], hydrocarbon stapled peptides [21], peptoids [22], terphenyls [23], β-peptides [24], among many others [25]. All of these compounds operate under a similar assumption that tight binding to HDM2 by a designed molecule will inhibit the interaction of HDM2 with p53, prevent p53 ubiquitination and degradation, and allow p53 to induce apoptosis and cell-cycle arrest in cancerous cells with an intact p53/HDM2 system. The protein p53 transcriptionally activates HDM2 in a feed-back loop and therapeutics that seek to activate p53 by binding HDM2 must overcome the increased levels of HDM2 that result from increased levels of p53. Huh7 cells are human hepatoma cells that express a mutant-p53 (Y220C/del genotype), which is the most common mutation found in p53 that is not located at the DNA binding interface [26], [27]. The p53-Y220C mutation has been structurally characterized and this mutation renders the protein temperature sensitive wherein the mutated p53 retains DNA binding ability at sub-physiological temperatures (24°C and 30°C) but does not transactivate gene expression at 37°C [28], [29]. This inability to transactivate at physiological temperatures interrupts the negative feedback loop between p53_Y220C_ and HDM2 and will allow us to observe the effects of compounds on levels of HDM2 without the interfering negative feedback loop.

In this report, we describe the synthesis and biological properties of a series of spiroligomers, which bind HDM2 in the cleft of the p53 transactivation domain. We further show that one spiroligomer, compound **1**, penetrates human liver cancer cells via passive diffusion and increases the levels of HDM2 *in vivo*.

## Results and Discussion

**Figure 1:**
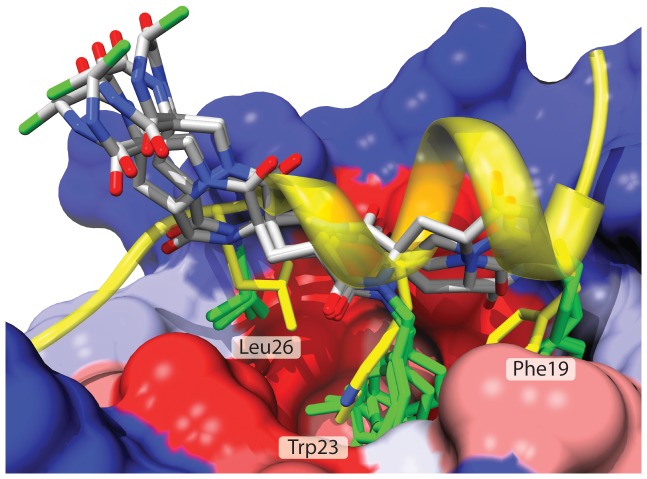
The modeled structures of the four diastereomeric spiroligomers 1, 8, 9, and 11 (white, blue, red and green tubes) superimposed onto the p53 helix (yellow ribbon) within the binding pocket of HDM2 (surface) showing the similar presentation of the functional groups (designated green), which can be tuned by changing the stereochemistry of the spiroligomer backbone.

As a first step to creating functionalized spiroligomers that modulate protein function we chose to create spiroligomers that could mimic the HDM2 bound conformation of p53. We have recently described versatile and efficient solution and solid-phase synthetic strategies for synthesizing functionalized spiroligomers [15], [16]. This methodology exploits our recent discovery of a new reaction, called “acyl-transfer coupling” that enables the formation of hindered tertiary amides and diketopiperazines by coupling active esters with unprotected *N*-alkyl amino acids [30]. To identify functionalized spiroligomers that could mimic the HDM2 bound conformation of p53 we used an in-house developed software package called CANDO [1] that rapidly builds models of millions of spiroligomers and scores them for their ability to display functional groups in desired constellations. CANDO is able to vary back-bone stereochemistry and conformations as well as side-chain conformations to identify spiroligomers that could present functional groups to mimic the presentation of the Cα-C_β_ bond vectors of residues Phe19, Trp23 and Leu26 of p53 bound to HDM2 from the X-ray crystal structure [3]. The scoring function used by CANDO in the design of spiroligomers was the least squares fit of the Cα-Cβ bond vectors of these residues superposed onto the bond vectors that we could functionalize on the spiroligomers. CANDO presented several stereoisomers out of 256 possible stereoisomers (eight stereocenters) that modeling suggested could mimic the N-terminal peptide of p53 including the stereoisomer with all (*S*) stereochemistry (Figure 1, Figure 3). We then synthesized on solid support compounds **3–7** and compounds **1** and **8–12**, two series of spiroligomers which explore the effects of varying functional groups on one stereochemically defined backbone (compounds **3–7**) and varying stereochemistry with one set of functional groups (compounds **1**, **8–12**) on the binding to HDM2 [15], [16].

**Figure 2:**
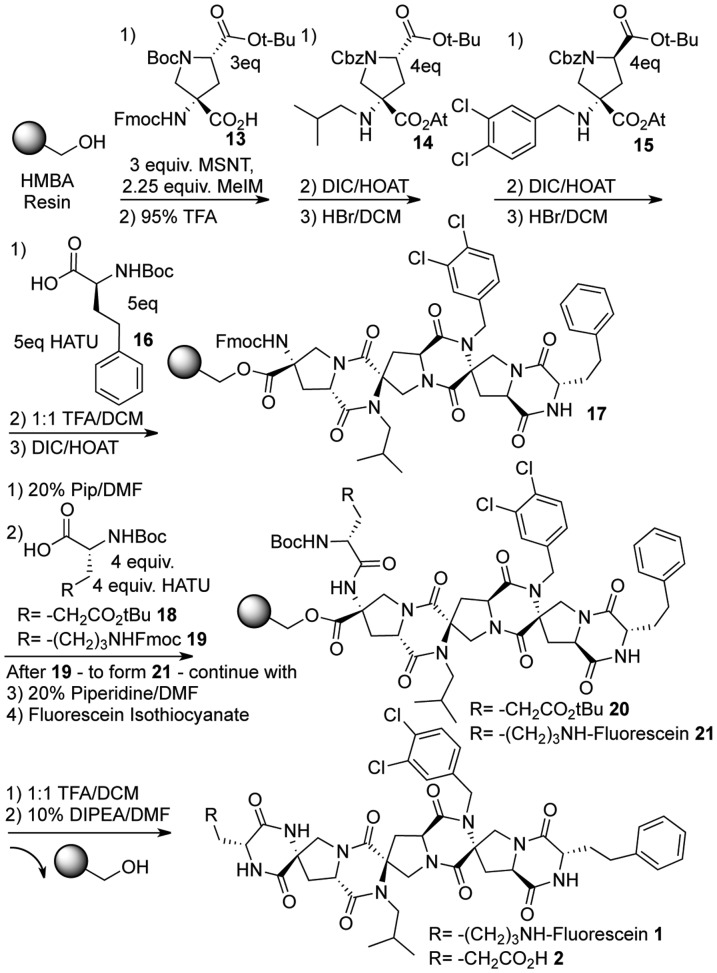
The solid-phase synthesis of spiroligomers 1 and 2 using the HMBA resin. Building blocks were successively coupled using either esterification conditions for residue **13** or conditions previously developed to achieve hindered active esters **14** or **15** [16]. Further derivatization of the oligomers followed by cyclization of the terminal DKP released spiroligomers **1** and **2** from the solid support [16].

The synthesis of compounds **1** and **2** is shown as a representative example and other spiroligomers were synthesized using the same synthesis as compound **1** with different monomers (Figure 2). The synthesis is modular like peptide synthesis and can be extended to incorporate more monomers and increase the contact area of functionalized spiroligomers with MDM2 without increasing flexibility of the molecules at the same time. The synthesis utilized the acid-stable hydroxy-methyl benzamide (HMBA) resin in order to be able to remove the acid-labile protecting groups on solid support. First, Fmoc-protected building block **13** was immobilized on the resin using the esterification reagent 1-(mesitylene-2-sulfonyl)-3-nitro-1,2,4-triazole (MSNT) in the presence of methyl-imidazole. This and all subsequent couplings were repeated to ensure quantitative acylation. The *tert*-butyloxycarbonyl (Boc) and *tert*-butyl ester protecting groups were then simultaneously removed using 95% trifluoroacetic acid (TFA) in the presence of the cation scavenger triisopropylsilane (TIS) to expose the next resin-bound prolinyl amino acid. The next building block was then activated with 1 equivalent of diisopropylcarbodiimide (DIC) in the presence of 6-fold excess of 1-hydroxy-7-azabenzotriazole (HOAt) to give the hindered amino-OAt ester **14**. These conditions have been shown to produce competent acylating species from hindered amino acid building blocks [15], [16]. The addition of activated building blocks such as **14** with prolinyl amino acids has been shown to produce mixtures of regioisomeric tertiary amides as well as the fully dehydrated diketopiperazine (DKP). The addition of another equivalent of DIC/HOAt to the resin converts all intermediate species into that of the desired DKP. The carbobenzyloxy (Cbz) protecting group and the *tert*-butyl ester protecting groups were then removed by treatment of the resin with 33% HBr in acetic acid to expose the next resin-bound prolinyl amino acid. The same procedure of acylation, DKP formation with DIC/HOAt, and then deprotection with HBr was then used to install the dichlorobenzyl residue **15**. The next residue, Boc-homophenylalanine **16**, was activated using HATU and 2 equivalents of diisopropylethylamine (DIPEA), followed by Boc removal with TFA and DKP formation using DIC/HOAt to yield resin-bound intermediate **17**. The fluorenylmethyloxycarbonyl (Fmoc) group was then removed with piperidine, the resin was split into two portions and one portion was acylated with *tert*-butyl protected glutamic acid **18** to produce resin bound oligomer **20**. The other portion was acylated with Fmoc protected lysine **19** followed by treatment with 20% piperidine to remove the Fmoc group and treatment of fluorescein isothiocyanate to yield resin-bound oligomer **21**. The final Boc deprotection of both oligomer **20** and **21** was then accomplished by addition of TFA, and neutralization of the resin induced diketopiperazine formation with concomitant release of the oligomer from the solid support to yield fully rigidified spiroligomers **1** and **2**.

**Figure 3:**
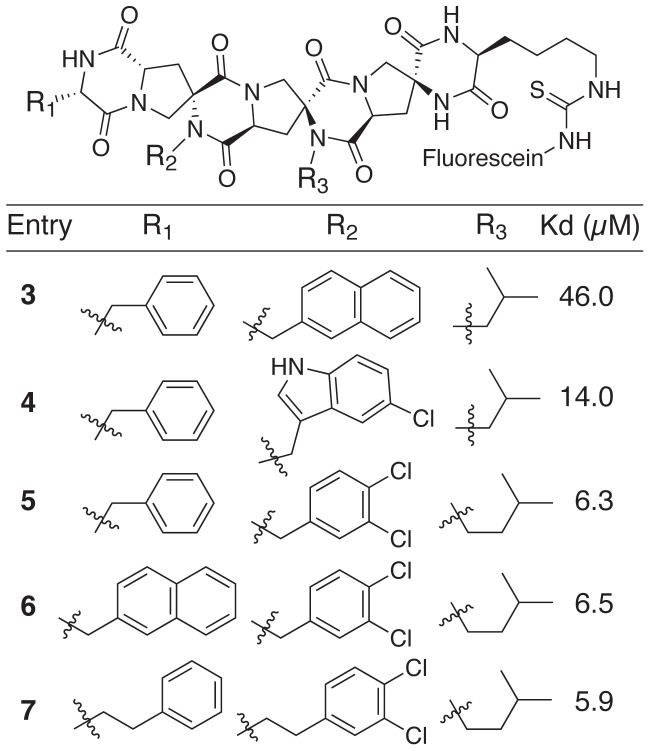
The structures of spiroligomers, which explored the use of different functional groups as side-chains on a fixed backbone (all centers have “*S*” stereochemistry).

To measure the binding affinity of the functionalized spiroligomers to HDM2 we utilized a direct binding fluorescence polarization assay. The binding of the fluorescein labeled spiroligomers was measured at 10 nM spiroligomer in the presence of varying concentrations of HDM2_25–117_ [31]. Under the conditions of this assay, a fluoresceinated derivative of the p53 peptide (at 10 nM) had a measured K_d_ of 0.62 µM, in close agreement with previously published results [21]. The initial screen of spiroligomers including **3–7** was carried out on the spiroligomer backbone containing all (*S*) stereochemistry to identify appropriate functional groups to mimic the side chains of Phe19, Trp23 and Leu26 of the p53 helix. This subset of the spiroligomers in this side-chain screen is shown in Figure 3. Changing either R_1_ or R_3_, the surrogates for the side chains of phenylalanine and leucine, had little effect on the binding of the spiroligomers. However, using different substituted aromatic functional groups (R_2_) in place of tryptophan showed an ∼8-fold improvement in binding by using the dichlorobenzyl group [32] (spiroligomers **5–7**) as a mimic of the p53 Trp23 indole ring.

**Figure 4:**
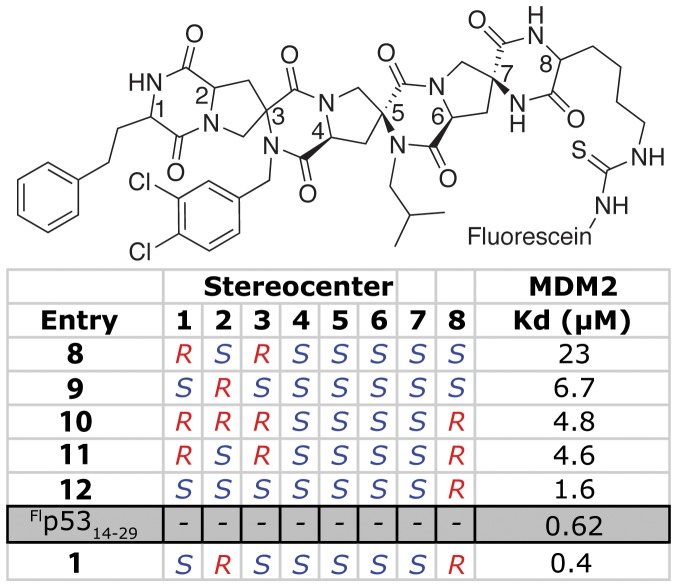
The stereoisomers 8–12 and 1 were suggested by CANDO as being able to mimic the presentation of the side-chains of p53 when bound to HDM2.

Once a set of reasonable mimics of the side chains was determined a second round of optimization was undertaken by changing the stereochemistry of the spiroligomer scaffold to the other stereoisomers suggested by CANDO while keeping the side-chains the same (Figure 1, Figure 4). Examination of the table shows that an ∼60-fold decrease in K_d_ was achieved by tuning the presentation of the three side chains by changing the stereochemistry of the spiroligomer backbone. Compound **1**, the tightest binding spiroligomer identified, had a K_d_ of 0.4 µM, and bound more tightly than the fluoresceinated derivative of p53 (^Fl^p53_14–29_). Noteworthy in this stereochemical series of spiroligomers is the subtle relationship between stereochemistry and affinity. For example, compounds **12** and **1** are epimers, with only a minor change in their relative orientation of their side chains by modeling, but they have a four-fold difference in binding. In contrast, the epimeric scaffolds **10** and **11** have nearly identical affinity for HDM2. Finally, spiroligomers **9** and **1** change only the stereocenter of the lysine residue to which the fluorophore is attached and display a nearly 17-fold increase in affinity. Modeling suggests that stereoisomer **1** projects the fluorescein side chain toward the protein surface while **9** projects it away, albeit far from the hydrophobic cleft normally associated with the binding of p53 to HDM2.

**Figure 5:**
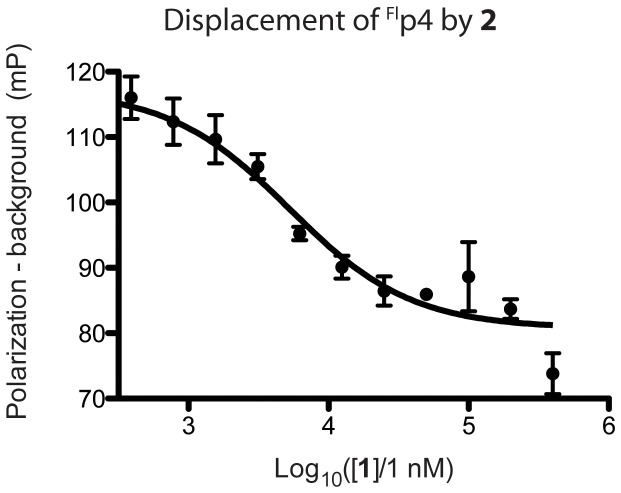
Fluorescence polarization measurement of ^Fl^p4 peptide (K_d_ of 0.16 µM with HDM2) being displaced by spiroligomer 2. The fit curve is consistent with a K_i_ value of 5 µM.

To test whether spiroligomers were binding into the hydrophobic cleft of HDM2, we undertook competition experiments with a low nanomolar binding fluoresceinated mutant of the p53 peptide called ^Fl^p4 (K_d_ is 0.16 µM for HDM2) [20]. Incubating ^Fl^p4 at 10 nM with 2 µM HDM2 and a series of concentrations of spiroligomer **2** which lacked the fluorescein group gave a K_i_ of 5 µM (Figure 5). These results suggest that the spiroligomer **2** lacking a fluorescein was able to compete with a fluorescein labeled p53-analog that is known to bind to the hydrophobic groove of HDM2. To demonstrate the selectivity of spiroligomer **1**, we undertook binding experiments with HDMX, a homolog of HDM2. Compound **1** had a measured K_d_ of 19 µM to HDMX, demonstrating that **1** is selective for HDM2 (data not shown).

**Figure 6:**
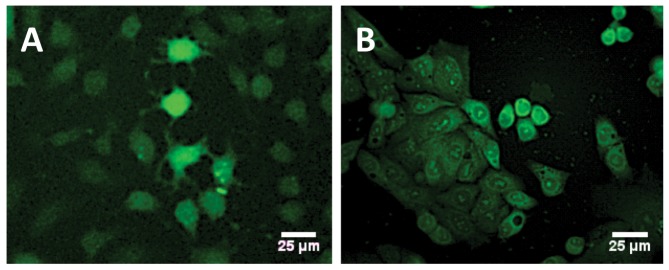
(A) Fluorescent imaging of spiroligomer 3 in Huh7 cells (spiroligomer 3 concentration 2 µM, incubated for 24 hours, washed with phosphate-buffered saline (PBS), followed by fixation of the cells. (B) HepG2 cells that were incubated with spiroligomer **1** (0.5 mM) for 15 hours, washed with PBS and then imaged with fluorescent microscopy (concentration 0.5 µM), incubated for 15 hours and then imaged.

**Figure 7:**
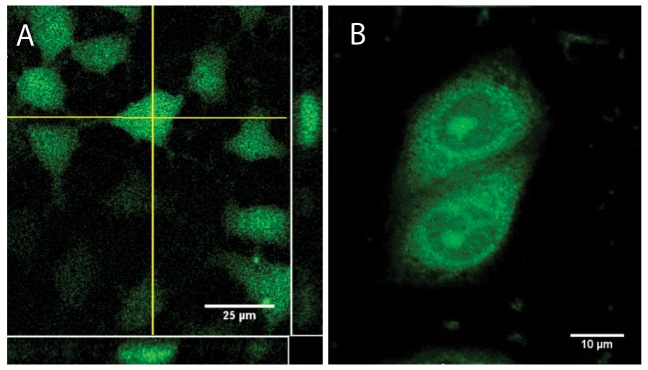
(A) Confocal imaging of spiroligomer 3 (2 µM 3, incubated for 24 hours and then washed with PBS) in live Huh7 cells. Yellow lines represent two orthogonal sections of a z-series showing the distribution of green fluorescence in the cells. (B) Optically sectioned HepG2 cells exposed to spiroligomer **1** (0.5 µM **1**, incubated for 15 hours and then washed with PBS) by confocal microscopy. Spiroligomer **1** is localized in both cytoplasmic and nuclear compartments.

**Figure 8:**
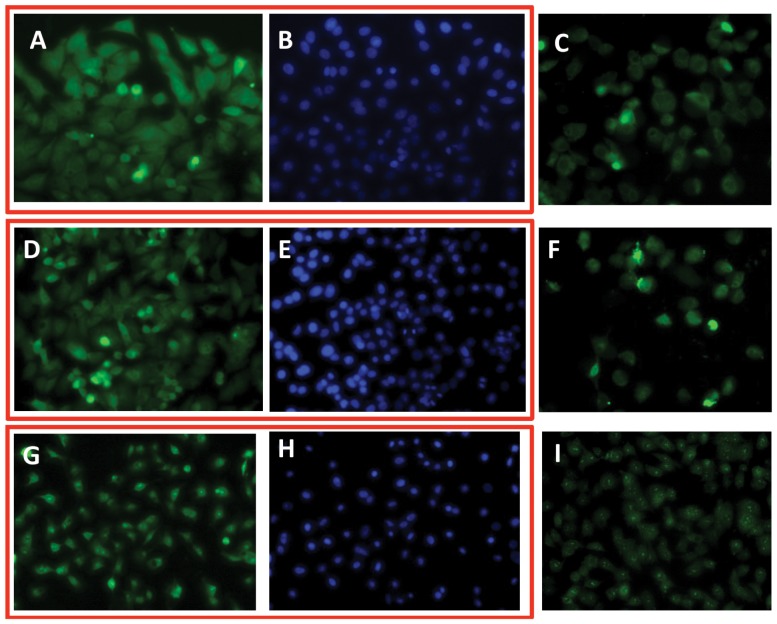
Fluorescence microscopy images for compound 1 (5 µM concentration) in HepG2 cells. (A) 37°C treated cells for 3.75 hours and then fixed and imaged; (B) DAPI stained cells shown in (A); (C) live cells treated with **1** for 3.75 hours at 37°C and then imaged; (D) Cells were pre-cooled at 4°C for 30 min and then treated with **1** for 3.75 hours and then washed, fixed and imaged; (E) DAPI stained cells shown in (D); (F) Live cells treated with **1** for 3.75 hours at 4°C (as in D); (G) Cells pre-treated with 10 mM NaN_3_ and 50 mM deoxy-D-glucose for 60 minutes and then with **1** for 3.75 hours and then washed, fixed and imaged; (H) DAPI stained cells shown in (G); (I) live cells treated with NaN_3_ and deoxy-D-glucose (as in G).

With the sub-micromolar HDM2 binding spiroligomer **1** in hand we sought to evaluate its effect in cell-culture. We first examined the cell-penetrating capability of two of the functionalized spiroligomers. Compound **3** was incubated with Huh7 cells (human hepatoma cells, mutant-p53 with a p53-Y220C/del genotype, purchased from ATCC (Manassas, VA)) for 24 hours at a concentration of 2 µM and observed with fluorescence microscopy (including confocal imaging), after washing away the media containing the fluorescein labeled spiroligomer using phosphate buffered saline (PBS). Significant uptake of **3** was seen as judged by the amount and fluorescent intensity of the cells, including distribution throughout the cytoplasm and nucleus as seen with confocal microscopy (Figure 6A). We then incubated spiroligomer **1**, with both Huh7 cells (not shown) and HepG2 (human liver hepatoblastoma cells, purchased from ATCC) [26] and imaged them (Figure 6B). Compound **1** at concentrations ranging from 2 µM to 40 µM had no visible effect on cell morphology or viability with Huh7, HepG2 or primary human myocytes (data not shown) and no visible cytotoxicity as observed using fluorescence microscopy after exposures of up to 72 hours. Confocal fluorescence images verified the distribution of compound **1** throughout the cytoplasm and nucleus (Figure 7). When the cells were incubated with fluorescein for similar times, no fluorescence uptake was observed (not shown). With this evidence of cellular uptake of fluorescently labeled spiroligomers we sought to determine if the uptake was through active transport or passive diffusion. HepG2 cells were pre-cooled for 30 min at 4°C in 475 µL of media and then 25 µL of spiroligomer **1** in phosphate buffered saline (pH 7.4) was added to the media to achieve a concentration of 5 µM of compound **1**. The cells were then incubated at 4°C for 3.75 hours and then the media with **1** was removed. Fluorescence imaging showed substantial uptake of fluorescence that was indistinguishable from control cells treated with **1** at 37°C (Figure 8). In further experiments, HepG2 cells at 37°C were pre-treated with 10 mM sodium azide together with 50 mM deoxy-*D*-glucose (60 minutes pre-treatment) followed by addition of compound **1**. These cells also showed uptake of fluorescence at levels that were indistinguishable from control cells treated with **1** at 37°C (Figure 8G&I). Incubation of both Huh7 and HepG2 cell lines with fluorescein at 37°C as a control produced no visible uptake of fluorescence. Despite having molecular weights in excess of 1,300 Daltons, both compound **1** and **3** penetrate human cells and compound **1** penetrates cells when active transport is inhibited suggesting that these compounds penetrate cells by passive diffusion. The lack of flexibility and the fact that five of the eight amides of these compounds are *N*-alkylated may contribute to their good bioavailability despite their large size [33].

**Figure 9:**
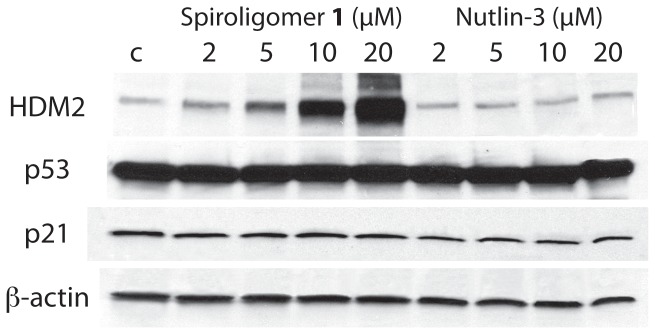
Western blot analysis of HDM2, p53 and p21 in Huh7 cells as a function of concentration of spiroligomer 1 (left) or Nutlin-3 (right).

**Figure 10:**
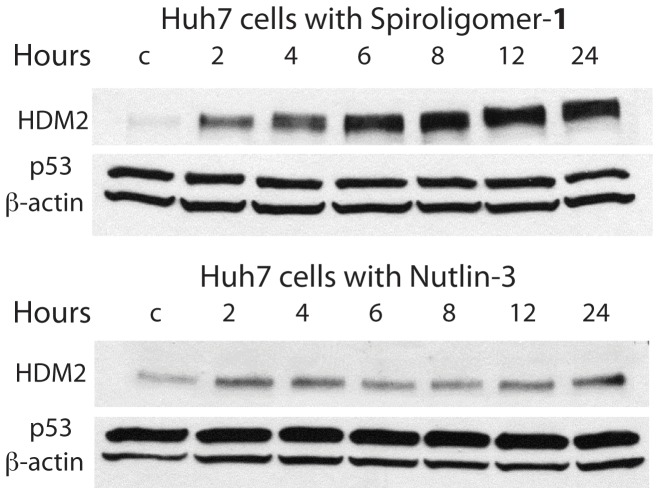
Time dependent Western analysis of the effect of compound 1 (15 µM) and nutlin-3 (15 µM) with Huh7 cells. Compound **1** causes a large increase in HDM2 levels with no effect on p53_Y220C_ levels. Nutlin-3 has very little effect on HDM2 and no observable effect on p53_Y220C_ levels.

**Figure 11:**
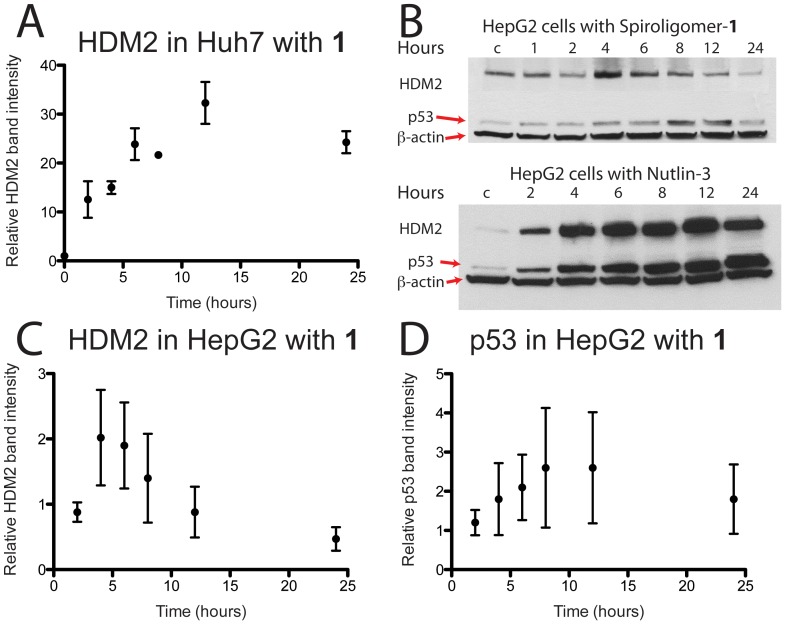
Time dependent Western analysis of spiroligomer 1 (15 µM) and Nutlin-3 (15 µM) added to Huh7 and HepG2 cells. (A) Relative HDM2 band intensity obtained when compound **1** was incubated with Huh7 cells. This plot is the average of two gels and shows a greater than 30-fold increase in the levels of HDM2. (B) Representative western gels showing the effect on HDM2 and wt-p53 in HepG2 cells in the presence of compound **1** and Nutlin-3. (C) Plotted average HDM2 band intensity relative to control (intensity of 1) as a function of time of HepG2 cells incubated with compound **1** at 15 µM. (D) Plotted average of p53 band intensity relative to control (intensity of 1) as a function of time of HepG2 cells incubated with compound **1** at 15 µM. In (C) and (D) the results of six gels (see supporting information) are averaged and standard deviations are plotted on the data. Compound **1** leads to a transient increase in HDM2 levels at 4 hours followed by a decrease. The level of p53 also increases transiently and then decreases again and the p53 changes trail the HDM2 response.

Huh7 cells were incubated with spiroligomer **1** and Nutlin-3 in media at 2, 5, 10 and 20 µM for 17 hours at 37°C. Western blot analysis showed the surprising result that HDM2 levels in the cells treated with **1** increased more than 30-fold relative to control cells in a dose-dependent manner while p53_Y220C_ levels and p21 levels (a protein that is transcriptionally activated by p53) did not change (Figure 9). As a control experiment Huh7 cells were treated with Nutlin-3 at the same concentrations and duration and Nutlin-3 shows a very weak stabilization effect on HDM2. No visible effect on the levels of p53_Y220C_ was seen with either compound **1** or Nutlin-3 in Huh7 cells, suggesting that this temperature sensitive p53_Y220C_ mutant is neither ubiquitinated nor negatively regulated by HDM2. Quantitative real-time PCR analysis of Huh7 cells treated with spiroligomer **1** showed no effect of **1** on the level of mRNA for HDM2 (see supporting information). Huh7 cells were then incubated with **1** at 15 µM and cells were harvested at 2, 4, 6, 8, 12 and 24 hours. Western blot analysis of these cells showed a large increase in HDM2 concentration with time and no change in p53_Y220C_ levels with time (Figure 10, Figure 11A). When the same experiment was carried out using Nutlin-3 at 15 µM only very small changes in the levels of HDM2 relative to the β-actin loading control were seen with time. Our hypothesis is that compound **1** stabilizes HDM2 to proteolysis. Studies are underway to determine the mechanism of HDM2 stabilization by compound **1**.

Time dependent Western analysis of the effect of spiroligomer **1** on the levels of HDM2 and wt-p53 in HepG2 cells were also carried out. In HepG2 cells the HDM2 levels increased on average 2-fold at 4–8 hours after introduction of compound **1** and then decreased after that (Figure 11B&C). The wt-p53 increased on average about 2–3 fold at 6–12 hours and then decreased after that (Figure 11B&D). This is in contrast to the well-known effect of Nutlin-3 observed in many cell lines in which the level of wt-p53 increases in a dose-dependent manner with a concomitant increase in the levels of HDM2 (Figure 11B) [18], [34]. In HepG2 cells, the HDM2 stabilization of compound **1** seen in Huh7 cells is possibly muted by the negative feedback loop of p53 and HDM2. The weak, transient stabilization of p53 in HepG2 cells by compound **1** may be due to its dissociation constant with HDM2 (400 nM) being on par with the affinity of p53 for HDM2.

## Conclusions

Two series of functionalized spiroligomers were synthesized that screened side-chain compatibility and backbone stereochemistry to mimic the conformation of p53 bound to HDM2. The spiroligomer **1** was developed to demonstrate that spiroligomers can be designed to bind proteins and to begin evaluating their drug properties. Spiroligomer **1** binds HDM2, penetrates human cells by passive diffusion and has the unexpected biological effect of stabilizing HDM2 in cell culture. These early results suggest that spiroligomers, despite their size, can penetrate cells and can be developed to bind protein surfaces and invoke a biological response. To the best of our knowledge, the stabilization of HDM2 by **1** is a novel phenomenon that warrants further study because it may be useful as a chemical tool to elucidate how HDM2 is regulated in cells [35]. Further studies are underway to elucidate the mechanisms of HDM2 stabilization.

## Supporting Information

Supporting Information S1 **ynthesis and characterization of the helix mimic spiroligomers.** Details of the binding experiments as well as the cell culture and Western blot analysis. The full reference #5.
(PDF)
