# Supplementary material for: A Spiroligomer α-Helix Mimic That Binds HDM2, Penetrates Human Cells and Stabilizes HDM2 in Cell Culture
Source: PLoS One. 2012 Oct 18;7(10):e45948. doi: 10.1371/journal.pone.0045948 (PMC3475717; doi:10.1371/journal.pone.0045948)
Supplement: Supporting Information S1 — ynthesis and characterization of the helix mimic spiroligomers. Details of the binding experiments as well as the cell culture and Western blot analysis. The full reference #5. (PDF) [file pone.0045948.s001.pdf]

Supporting Information for:

A Spiroligomer α-Helix Mimic that Binds HDM2, Penetrates Human Cells and Stabilizes HDM2 in Cell Culture

Zachary Z. Brown, Kavitha Akula, Alla Arzumanyan, Jennifer Alleva,
Marcus Jackson, Eugene Bichenkove, Joel B. Sheffield,
Mark A. Feitelson, Christian E. Schafmeister*
*To whom correspondence should be addressed; email: meister@temple.edu

Supporting Information Table of Contents

General Methods....................3
General Synthetic Procedures....................3
Solid Phase Synthesis of Spiroligomer Helix Mimics....................4
HDM2 Protein Expression and Purification....................9
Direct Binding Fluorescence Polarization Experiments....................11
Competition Fluorescence Polarization Experiments....................13
Cell Culture and Treatment....................14
Fluorescence and Confocal Microscopy....................14
Western Blotting and PCR analysis....................15
Active vs. Passive Transport Experiments....................16

Figure **S.1** HPLC trace of crude cleavage of spiroligomer **1**....................6
Figure **S.2** HPLC trace of purified spiroligomer **1**....................6
Figure **S.3**. HPLC trace of purified spiroligomer **2**....................7
Figure **S.4**. Direct binding plot of spiroligomer **2**....................12
Figure **S.5**. Western blot analysis of spiroligomer **1** and Nutlin-3....................15
Figure **S.6**. Western Blot replicates of compound 1 with HepG2 cells....................16
Figure **S.7**. PCR analysis of spiroligomer **1** in Huh7 cells....................17
Figure **S.8**. ^{1}H NMR analysis of spiroligomer **1**....................19
Figure **S.9**. HMQC NMR analysis of spiroligomer **1**....................20
Figure **S.10**. HMBC NMR analysis of spiroligomer **1**....................21

Table **S.1**. Functional groups of the structural analogs of spiroligomer....................8
Table **S.2**. LC-MS characterization data for the spiroligomer **3-7** and **S10-S13**....................8
Table **S.3**. LC-MS characterization data for the spiroligomer **8-12**....................8
Table **S.4**. Raw values for the polarization plot in Figure **S.4**....................11

Table **S.5**. Parameters used for the fitting of Figure **S.4**...12
Table **S.6**. Raw values for the competition polarization plot in Figure **S.5**.................................13
Table **S.7**. Parameters used for the fitting of Figure **S.5**...14

General Methods. Anhydrous dichloromethane (DCM), anhydrous dimethylformamide (DMF), anhydrous methanol (MeOH), HBr (33% in glacial AcOH), redistilled diisopropylethylamine (DIPEA) and Nutlin-3 were obtained from Sigma-Aldrich and used without purification. Pd/C was obtained from Strem Chemicals. All amino acids and HMBA-AM resin were obtained from either Novabiochem or Bachem. HOAt was obtained from Genscript. All other reagents were obtained from Sigma-Aldrich and used without further purification.

HPLC-MS analysis was performed on a Hewlett-Packard Series 1200 with a Waters Xterra MS C18 column (3.5 μm packing, 4.6 mm x 150 mm) with a solvent system of H_2O/acetonitrile with 0.1% formic acid at a flow rate of 0.8 mL/min. NMR experiments were performed on a Bruker Advance 500mHz NMR; NMR chemical shifts (δ) reported relative to DMSO-d_6 residual solvent peaks. When possible, rotamers were resolved by performing the analysis at 365 K. Assignment of 2D NMR data was performed using Sparky 3 (T. D. Goddard and D. G. Kneller, University of California, San Francisco).

RP-purification was performed on an ISCO (Teledyne, Inc.) automated flash chromatography system with a RediSep R_f-12 gm RP column or on a Varian Prostar Prep HPLC with a Waters Xterra column (5 μm packing, 19 mm x 100 mm) with a solvent system of H_2O/acetonitrile with 0.1% formic acid at a flow rate of 18 mL/min. HRMS analysis was performed by Ohio State University Proteomics Research Facility (ToF/ES).

General Procedure for the Preparation of Functionalized Building Blocks. The functionalized *pro4* derivatives were prepared from the corresponding *pro4* amino acids using the method of reductive alkylation has been described previously.[1-3]

General Procedure for Removal of Boc and *t*-Butyl Groups. The Boc and *t*-Butyl ester protecting groups were simultaneously cleaved from the resin bound oligomer by treatment with 95% trifluoroacetic acid (TFA) with 5% triisopropylsilane (TIS) used as a scavenger. The deprotection was allowed to proceed for one hour, washing the resin 5x with DCM, and then the deprotection was repeated for an hour. The resin was then washed 5x with DCM, 5x with DMF, and neutralized with 5% DIPEA in DMF.

General Procedure for Removal of Cbz and *t*-Butyl Groups. The Cbz and *t*-Butyl ester protecting groups were simultaneously cleaved from the resin bound oligomer by treatment with 1:1 33% HBr/AcOH in DCM. The deprotection was allowed to proceed for 30 minutes, washing

the resin 5x with DCM, and then the deprotection was repeated for 30 minutes. The resin was then washed 5x with DCM, 5x with DMF, and neutralized with 5% DIPEA in DMF.

General Procedure for Activation and Coupling. The activation and coupling method of functionalized spiroligomers has been described previously.[1,2] The building block to be activated (3 eq relative to resin loading) was suspended in a 1:2 mixture of DMF:DCM (conc. of 50 mM) followed by HOAT (6 eq relative to amino acid). With stirring, the diisopropylcarbodiimide (DIC, 1 eq relative to amino acid) was then added and the activation allowed to proceed for 1.5 hours at room temperature. The resin was then suspended in a minimal amount of DMF (~200 μL), and DIPEA (2 eq relative to resin loading) was added. The preactivated amino-OAt ester was then added in a single portion and allowed to react for the specified amount of time. The resin was then washed 3x with DCM and then 3x with DMF. The resin was then treated with an additional aliquot of DIC (5 eq relative to resin loading) and HOAT (5 eq relative to resin loading) in a 1:2 DCM:DMF mixture and allowed to react for 1 hour to convert any of the single amide product into the corresponding diketopiperazine.

General Procedure for Removal of Fmoc Group. Fmoc deprotection was conducted by treatment of the resin with 20% piperidine in DMF for 5minutes, washing the resin 5x with DMF, treatment with 20% piperidine in DMF for 15 minutes, and finally washing the resin 5x with DMF.

Solid Phase Synthesis of Spiroligomer Helix Mimics. The solid-phase synthesis of functionalized spiroligomers has been recently published.[2] The solid-phase synthesis of fluoresceinated compound **1** (compound, K_d=400 nM) and the competition experiment spiroligomer (compound **2**, K_i=5 μM against the P4 peptide) are detailed below as representative examples. All other syntheses of helix mimics follow a similar format using the corresponding building blocks. The syntheses below as well as all other spiroligomer syntheses used double coupling to ensure quantitative reactions. The LC-MS characterization for all oligomers is shown in the Tables that follow.

Solid Phase Synthesis of Compounds 1 & 2. 75 mg of HMBA-AM resin (1.1 mmole/gm resin loading, 83μmole) was charged to an 8 mL solid phase reactor and agitated using a magnetic stir bar and thoroughly washed with DCM. The first building block, compound **13**, (247 μmole, 137 mg), MSNT (1-(2-Mesitylenesulfonyl)-3-nitro-1H-1,2,4-triazole, 247μmole, 73mg) and *N*-methyl-imidazole (186 μmole, 15 μL) were dissolved in 1.2 mL of DCM (anhydrous), added to

the resin, and allowed to react for 2 hours. The building block was then deprotected with 1.5 mL of 95% TFA using the “General Procedure for Removal of Boc and *t*-Butyl Groups”. The next building block, compound **14** (247 μmole, 104 mg), was activated and coupled for 3 hours using the “General Procedure for Bis-Amino Acid Activation and Coupling” followed by deprotection using the “General Procedure for Removal of Cbz and t-Butyl Groups”. The next building block, compound **15** (247 μmole, 129 mg), was activated and coupled for 3 hours using the “General Procedure for Bis-Amino Acid Activation and Coupling” followed by deprotection using the “General Procedure for Removal of Cbz and *t*-Butyl Groups”. Boc-(L)-HomoPhe-OH, compound **16** (413 μmole, 115 mg) and HATU (413 μmole, 157 mg) were combined in DMF (2.1 mL, conc. of 200 mM); DIPEA (825 μmole, 144 μL) was added and the reaction mixture allowed to sit for 10 minutes. The preactivated species was then added to the resin and allowed to react for one hour, followed by thorough washing of the resin with 5x DMF, and then 5x with DCM. The Boc group was then removed with 1.5 mL of 1:1 TFA in DCM for 20 minutes, followed by washing of the resin with 5x DCM and repeating the Boc deprotection treatment. The resin was then washed 5x with DCM, 5x with DMF, and neutralized with 5% DIPEA in DMF. A ninhydrin test was positive, which indicated the presence of a primary amine. The resin was then treated with an additional aliquot of DIC (5 eq relative to resin loading) and HOAT (5 eq relative to resin loading) in a 1:2 DCM:DMF mixture and allowed to react for 1hour. A subsequent ninhydrin test was negative, which indicated the diketopiperazine had been closed and the secondary amide had been formed. The resin was then washed 5x with DCM and 5x with DMF. The Fmoc group was removed using the “General Procedure for Removal of Fmoc Group”.

Solid Phase Synthesis of Fluoresceinated Spiroligomer (Compound 1, K_d=400 nM). Boc-(D)-Lys(Fmoc)-OH, compound **19** (413 μmole, 193 mg) and HATU (413 μmole, 157 mg) were combined in DMF (2.1 mL, conc. of 200 mM); DIPEA (825 μmole, 144 μL) was added and the reaction mixture allowed to sit for 10 minutes. The preactivated species was then added to the resin and allowed to react for 1 hour, followed by thorough washing of the resin with 5x DMF, and then 5x with DCM. The Fmoc group was then removed from the lysine side chain using the “General Procedure for Removal of Fmoc Group”. The fluorescein label was then attached using fluorescein 6-isothiocyanate (247 μmole, 96 mg) in DMF (2.1 mL, conc. of 200 mM) with DIPEA (247 μmole, 43 μL) overnight followed with thorough washing of the resin with DMF and then DCM. The Boc group was then removed with 1.5 mL of 1:1 TFA in DCM for 20 minutes, followed by washing of the resin with 5x DCM and repeating the Boc deprotection treatment. The oligomer was then cleaved from the resin with 10% DIPEA in DMF (total of 1.5 mL of

solution) overnight at room temperature. LC-MS analysis, See Figure **S.1** for crude LC-MS trace (5-95% H_2O/ACN with 0.1% formic acid) for oligomer (**1**): t_r=21.3 min., calced. for product (**1**)+H^+= 1307.4; found: 1307.2. The crude product was then RP purified (see Figure **S.2**) and lyophilized. Anal. Calced for $C_{66}H_{64}Cl_2N_{10}O_{13}S+Na^+$: 1329.3650, Found: 1329.3607 (difference 3.2 ppm). See Figure **S.7-S.9** for NMR characterization.

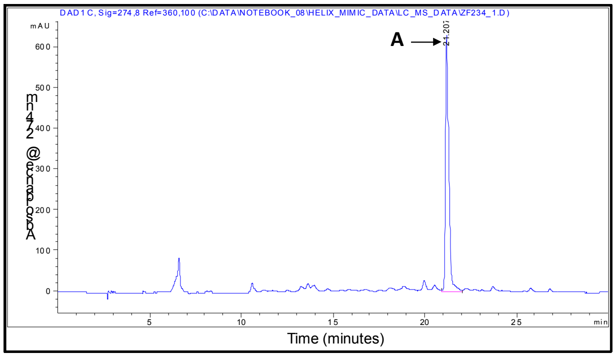

Figure **S.1**. HPLC trace of crude cleavage of bis-peptide **1**, monitoring at a wavelength of 274 nm with a gradient of 5-95% ACN/H_2O with 0.1% formic acid over 30 minutes. The peak marked "A" has a m/z = 1307.2 (calced for oligomer **1** + H^+: 1307.4).

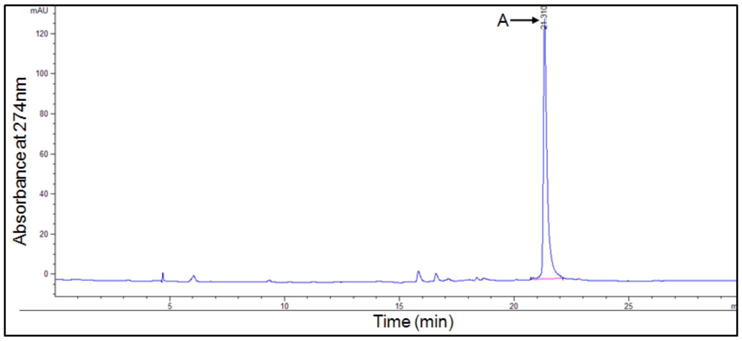

Figure **S.2** HPLC trace of purified bis-peptide **1**, monitoring at a wavelength of 274 nm with a gradient of 5-95% ACN/H_2O with 0.1% formic acid over 30 minutes. The peak marked "A" has a m/z = 1307.2 (calced for oligomer **1** + H^+: 1307.4).

.

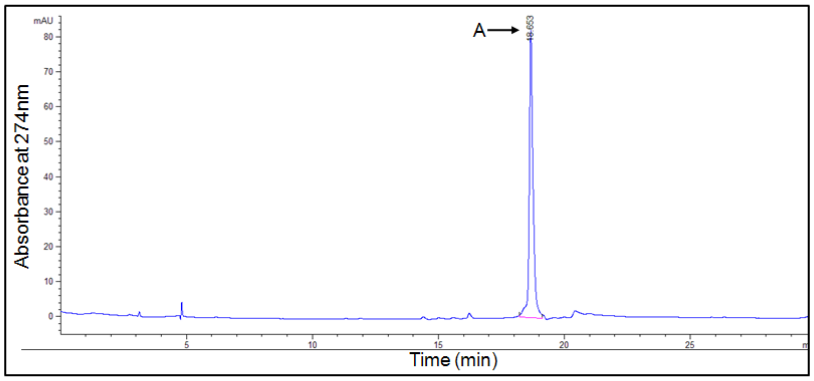

Figure **S.3**. HPLC trace of purified spiroligomer **2**, monitoring at a wavelength of 274 nm with a gradient of 5-95% ACN/H_2O with 0.1% formic acid over 30 minutes. The peak marked "A" has a m/z = 919.2 (calc'd for bis-peptide **2** + H^+: 919.3).

Solid Phase Synthesis of Spiroligomer (Oligomer 2, K_i=5.0 μM). The solid phase synthesis was continued from that of the intermediate oligomer above using 50mg of resin. The Fmoc group was removed using the "General Procedure for Removal of Fmoc Group". Boc-(D)-Glu(OtBu)-OH, compound **18**, (413 μmole, 125 mg) and HATU (413 μmole, 157 mg) were combined in DMF (2.1 mL, conc. of 200 mM); DIPEA (825 μmole, 144 μL) was added and the reaction mixture allowed to sit for 10 minutes. The preactivated species was then added to the resin and allowed to react for 1hour, followed by thorough washing of the resin with 5x DMF, and then 5x with DCM. The resin was then deprotected with 1.5 mL of 95% TFA using the "General Procedure for Removal of Boc and t-Butyl Groups". The resin was thoroughly washed with DCM and DMF, followed by treatment of the resin with 30% diethylamine in ACN for 2 hours at room temperature. LC-MS analysis, (5-95% H_2O/ACN with 0.1% formic acid). Oligomer (**2**): t_r=18.6 min., calced. for product (**2**)+H^+= 919.3; found: 919.2. The solvent was then removed *in vacuo*, the residue suspended in H_2O/ACN with 0.1% formic acid, RP-HPLC purified (see Figure **S.3**), and the desired fractions pooled and lyophilized. Anal. Calced for $C_{44}H_{48}Cl_2N_8O_{10}$+$Na^+$: 941.2768, Found: 941.2729 (difference 4.1 ppm).

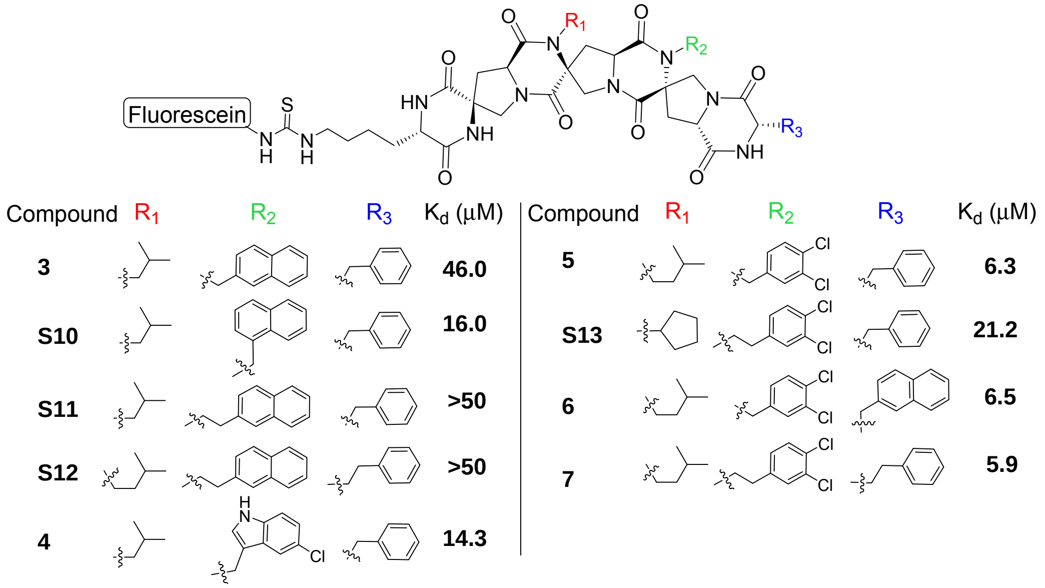

Table **S.1**. Functional groups used for the structural analog scan of spiroligomers binding to HDM2. The dissociation constants are given in μM; all scaffolds have the homochiral "S" stereochemistry in the backbone.

Compound	Retention Time (min)	Expected Mass + H^+	Found Mass
**3**	20.4	1275.4	1275.3
**S10**	20.7	1275.4	1275.2
**S11**	20.8	1289.5	1289.2
**S12**	22.1	1317.5	1317.1
**4**	19.8	1298.4	1298.1
**5**	21.3	1307.4	1307.1
**S13**	21.4	1319.4	1319.2
**6**	21.2	1357.4	1357.2
**7**	22.3	1335.4	1335.2

Table **S.2**. LC-MS characterization data for the spiroligomers **3-7** and **S10-S13**, which explored the use of different functional groups on HDM2 binding.

Compound	Retention Time (min)	Expected Mass + H^+	Found Mass
**8**	21.3	1307.4	1307.2
**9**	21.4	1307.4	1307.3
**10**	21.2	1307.4	1307.1
**11**	21.2	1307.4	1307.6
**12**	21.3	1307.4	1307.8
**1**	21.3	1307.4	1307.2

Table **S.3**. LC-MS characterization data for the spiroligomers **1** and **8-12,** which explored the effect of just stereochemistry of the scaffold on HDM2 binding.

HDM2 Protein Expression. A fusion protein consisting of residues 1-140 (the plasmid was a generous gift from the lab of Neil Zondlo, University of Delaware) was prepared by over-expression.[4] Plasmids were used to transform chemically competent E-coli BL21 Cells (Novagen), and a single colony was used to inoculate 2×4 mL LB media culture tubes containing 0.1 mg/mL ampicillin, at 37° C overnight. These overnight cultures were used to inoculate a 1 L culture of TB media containing 0.1 mg/ml ampicillin. The culture was incubated at 37° C with shaking at 250 rpm until the optical density at 600 nm reached to 0.9 absorbance units. Protein overexpression was then induced by the addition of 20% isopropyl β-D-thiogalactoside (IPTG) to 0.4 mM final concentration. The culture was then grown for additional 5 hours at 30° C. Then cells were harvested at 4° C by centrifugation at 4500 rpm for 20 min. The cell pellet was resuspended in chilled 1x Binding Buffer (0.5 M NaCl, 20 mMTris-Hcl, 5 mM imidazole, pH 7.9), frozen and stored at -80° C until protein purification.

Protein Purification. The frozen cell pellet was thawed at 4° C and lysed by sonication for 2 minutes with 10 sec on and 20 sec off. Cell debris was pelleted by centrifugation for 30 min at 15000 g, 4° C and the supernatant was filtered through a 0.2 μm syringe filter. A column was prepared using 2 mL Novagen His-Bind suspended resin and resin was washed with 3 volumes DI water. The flow rate maintained was not more than 0.5 ml/min throughout the purification. The resin was charged with 5 vol 1x Charge Buffer (50 mM $NiSO_4$) and then equilibrated with 3 vol 1x Binding Buffer (5 mM imidazole). After loading the column with prepared extract, the resin was washed with 10 vol 1x Binding Buffer and then with 6 vol 1x Wash Buffer (10 mM imidazole). Protein was then eluted with 6 vol 1x Elution Buffer (1 M imidazole). The eluate was captured in fractions of 0.5 ml. Fractions were assayed with Coomasie blue and desired fractions were combined and dialyzed using spectra/por 6 Dialysis Membranes of 10,000 MWCO (Spectrum Laboratories) in PBS (pH 7.4, 5 mM EDTA and 0.5 mM DTT) for overnight. The resulting protein was characterized by SDS-PAGE (4-20% gradient gel) for purity analysis and concentrations were measured by UV analysis. Experimental Protein yields were typically 1.7 mg from 1 L culture. Fresh protein was used immediately for protein binding experiments.

Synthesis of ^{Fl}p53 Control Peptide. A control peptide corresponding to residues 14-29 was synthesized on an Apex 396 multiple peptide synthesizer (aapptec, Louisville, KY). Rink Amide-AM resin, HATU activation and 30 minute double couplings were used for all residues. A β-Ala was installed on the N-terminus followed by fluorescein labeling by exposure of the peptidyl resin to 5 eq of FITC and 5 eq of DIPEA overnight. The primary sequence of the peptide was:

Fluorescein-β–Ala-L-S-Q-E-T-F-S-D-L-W-K-L-L-P-E-N-NH_2. The product was cleaved from the resin by treatment with 2 mL of 95% TFA/2.5%H_2O/2.5%TIS for 2 hours followed by concentration of the cleavage solution with a rotovap. The residue was then suspended in H_2O/ACN and the crude material RP-HPLC purified, and the desired fractions pooled and lyophilized. LC-MS analysis, (5-95% H_2O/ACN with 0.1% formic acid). $p53^{Fl}$ Peptide (^{Fl}**p53**): t_r=15.7 min., calced. for peptide(^{Fl}**p53**)+H^+= 2379.1; found: 2379.7.

Synthesis of ^{Fl}p4 Control Peptide. A control peptide called P4 was identified by the Holak group using phage display[5] as a very tight inhibitor of HDM2 was synthesized by the Temple University Solid-Phase Peptide Synthesis Facility on an CEM Liberty peptide synthesizer (CEM Corp, Matthews, NC). A β-Ala was installed on the N-terminus followed by fluorescein labeling by exposure of the peptidyl resin to 5 eq of FITC and 5 eq of DIPEA overnight. The primary sequence of the peptide was: Fluorescein-β–Ala-L-T-F-E-H-Y-W-A-Q-L-T-S-OH. The product was cleaved from the resin by treatment with 2 mL of 95% TFA/2.5%H_2O/2.5%TIS for 2 hours followed by concentration of the cleavage solution using reduced pressure. The residue was then suspended in H_2O/ACN and the crude material RP-HPLC purified, and the desired fractions pooled and lyophilized. LC-MS analysis, (5-95% H_2O/ACN with 0.1% formic acid). $P4^{Fl}$ Peptide (^{Fl}**p4**): t_r=15.1 min., calced. for peptide(^{Fl}**p4**)+H^+= 1955.8; found: 1955.7.

Fluorescence Polarization Experiments. Binding experiments were performed in a black 96-well Costar plate and used an Analyst GT plate reader (Molecular Devices, Sunnyvale, CA). The binding buffer used for all experiments was PBS (pH 7.4) with 5 mM EDTA and 0.5 mM DTT. All plates were read using the fluorescein excitation filter of 485 nm and the emission filter of 530 nm.

Direct Binding Fluorescence Polarization Experiments. Fluorescence polarization experiments were undertaken to assess the ability of spiroligomers to bind HDM2, a protein with hydrophobic cleft that binds the p53 N-terminal helical domain. Fluoresceinated oligomers (conc. of 10 nM) were incubated for 30 minutes at room temperature with sequential dilutions of hDM2 protein (final conc. 42 μM to 0.08 nM), followed by recording of the polarization data (see Figure **S.4**, and Tables **S.4** and **S.5**). The data was processed using the Graphpad Prism program[6] using a one-site, specific binding model.

[hDM2] (nM)	mx18 F.P. (millipolarization)		
42500	280.74	287.71	288.59
21250	289.83	291.58	284.9
10625	279.23	291.88	291.17
5312.5	283.09	283.22	294.56
2656.25	276.63	280.28	279.87
1328.125	249.64	254.41	260.31
664.0625	219.16	207.64	219.81
332.0313	176.96	168.11	165.21
166.0156	123.93	122.78	128.68
83.00781	90.07	92.68	90.31
41.50391	82.2	90.13	83.56
20.75195	75.45	72.99	76.91
10.37598	78.3	73.95	76.62
5.187988	68.52	66.82	65.52
2.593994	69.71	64.6	67.34
1.296997	74.46	61.79	70.28
0.6484985	76.88	65.12	66.54
0.3242493	75.62	76.93	62.45
0.1621246	65.92	65.99	65.14
0.08106232	68.58	68.27	70.47

Table **S.4**. Table of raw values used for the plot in Figure **S.4**; X values are HDM2 concentration (in nM) and Y values are polarization (in millipolarization units) for oligomer **1** (three independent replicates).

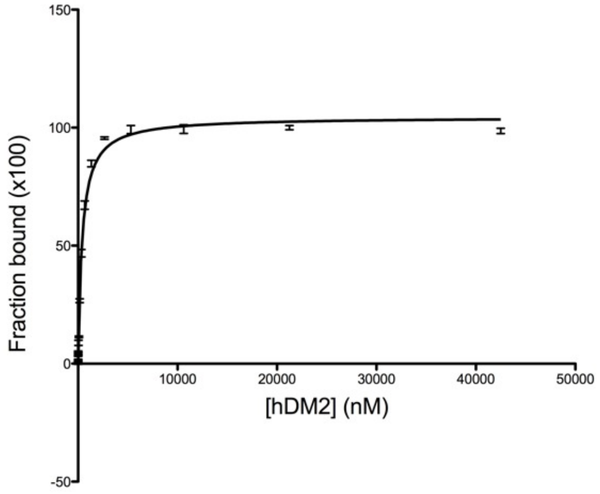

Figure **S.4**. Plot of log of hDM2 concentration (in nM) versus fraction bound for oligomer **1**.

Nonlin fit		A<br>mx18 F.P. (millipolarization)
1	One site -- Specific binding	
2	Best-fit values	
3	Bmax	104.5
4	Kd	402.9
5	Std. Error	
6	Bmax	1.016
7	Kd	19.24
8	95% Confidence Intervals	
9	Bmax	102.4 to 106.5
10	Kd	364.4 to 441.4
11	Goodness of Fit	
12	Degrees of Freedom	58
13	R square	0.9937
14	Absolute Sum of Squares	662.3
15	Sy.x	3.379
16	Number of points	
17	Analyzed	60

Table **S.5**. Parameters used for the non-linear fit for plot in Figure **S.4**.

Competition Fluorescence Polarization Experiments. To assess the ability of oligomer **2** to displace fluoresceinated p4 peptide (measured K_d=157 nM with respect to hDM2) from hDM2, competition experiments were undertaken. hDM2 (0.4 μM) and ^{Fl}**p4** control peptide (10 nM) were incubated at room temperature for 30 minutes in binding buffer, followed by the addition of serial dilutions of oligomer **2** (final conc. 400 μM to 0.194 μM). The binding experiments were incubated for a further 30 minute time period, followed by recording of the polarization data. See Tables **S.6** and **S.7.**

Bis-Peptide (nM)	Polar A (mP)		
399000	75.41	67.78	78.26
199500	84.38	85.9	80.84
99750	78.06	93.73	94.13
49875	86.21	84.81	86.83
24937.5	88.82	82	88.53
12468.75	91.7	86.62	91.97
6234.375	97.23	94.66	93.84
3117.188	103.88	103.29	109.28
1558.594	102.29	112.93	113.76
779.2969	107.07	119.05	110.95
389.6484	109.71	120.5	117.89
194.8242	115.56	119.19	107.52

Table **S.6**. Table of raw values used for the plot of the competition binding experiment; X values are oligomer **2** concentration (in nM) and Y values are polarization (in millipolarization units); data from three independent trials is shown.

Nonlin fit		A
		Polar A (mP)
1	One site - Fit Ki	
2	Best-fit values	
3	logKi	3.697
4	HotNM	= 10.00
5	HotKdNM	= 157.0
6	Bottom	80.75
7	Top	117.2
8	Ki	4972
9	Std. Error	
10	logKi	0.1354
11	Bottom	1.785
12	Top	2.309
13	95% Confidence Intervals	
14	logKi	3.421 to 3.972
15	Bottom	77.12 to 84.38
16	Top	112.5 to 121.9
17	Ki	2636 to 9379
18	Goodness of Fit	
19	Degrees of Freedom	33
20	R square	0.8670
21	Absolute Sum of Squares	955.1
22	Sy.x	5.380
23	Constraints	
24	HotNM	HotNM = 10.00
25	HotKdNM	HotKdNM = 157.0
26	Number of points	
27	Analyzed	36

Table **S.7**. Parameters used for the non-linear fit for the plot of the competition binding experiment.

Cell Culture and Treatment. HepG2 (expressing wild-type p53) and Huh7 (expressing mutant p53) cell lines were purchased from the American Type Culture Collection (ATCC) and cultured as monolayers in Dulbecco's modified Eagle's medium (DMEM) (Invitrogen) supplemented with 100 mM nonessential amino acid solution, 100 mM sodium pyruvate, 100 U/mL penicillin, 100 μg/mL streptomycin, and 10% heat inactivated fetal bovine serum (FBS) (all from Hyclone). Cells were maintained at 37° C and 5% CO_2 and were treated with media that contained 0.5% FBS. Nutlin-3 (Sigma) was dissolved in 0.01% DMSO (Sigma). This concentration of DMSO did not affect the cell phenotype. Both nutlin-3 and spiroligomers were added into the medium for final concentrations of 2 μM, 5 μM, 10 μM and 20 μM for 24 hours. For the western blot experiments detailed below, the time length for incubation with bis-peptide was 17 hours.

Fluorescence and Confocal Microscopy. Fluorescence and confocal microscopy were applied to live and fixed cells. Cells were grown on glass chambers and fixed with ice-cold 95:5 ethanol/acetic acid for 10 minutes at -20° C. Sections were washed with PBS and mounted in Vectashield aqueous mounting medium with DAPI (Vector laboratories). Fluorescent specimens were analyzed using an ECLIPSE Ti microscope (Nikon), lenses with hardened

filters (Nikon), and a Nikon DS-Fi1 camera, which was operated by NIS Elements computer software (Nikon). Confocal microscopy was performed with a Leica SP-1 microscope, with illumination at 488 nm and spectral detection.

Western Blotting. For protein extraction, cells were lysed in cell lysis buffer (Cell Signaling) with a protease inhibitor cocktail for 20 minutes on ice. Protein extracts (50 μg) were separated by SDS-PAGE electrophoresis, transferred to nitrocellulose membranes (Schleicher & Schuell), and incubated overnight with primary antibodies to p53, HDM2, p21, and β-actin (all from Santa Cruz Biotechnology). The blots were developed using ECL plus detection system (Amersham Biosciences) and exposed to Kodak imaging films (Kodak BioMax). Images were quantified using ImageJ software (NIH).

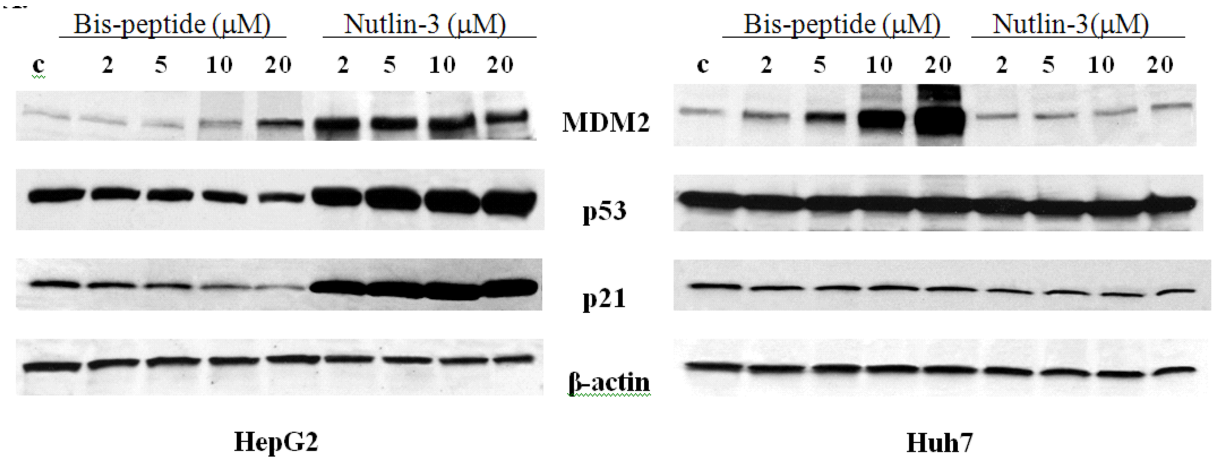

Figure **S.5**. Detection of bis-peptide **1** and Nutlin-3 in HepG2 (left) and Huh7 (right) cells. The concentration of each compound is given in μM and the three proteins (HDM2, p53 and p21) bands are labeled for each gel.

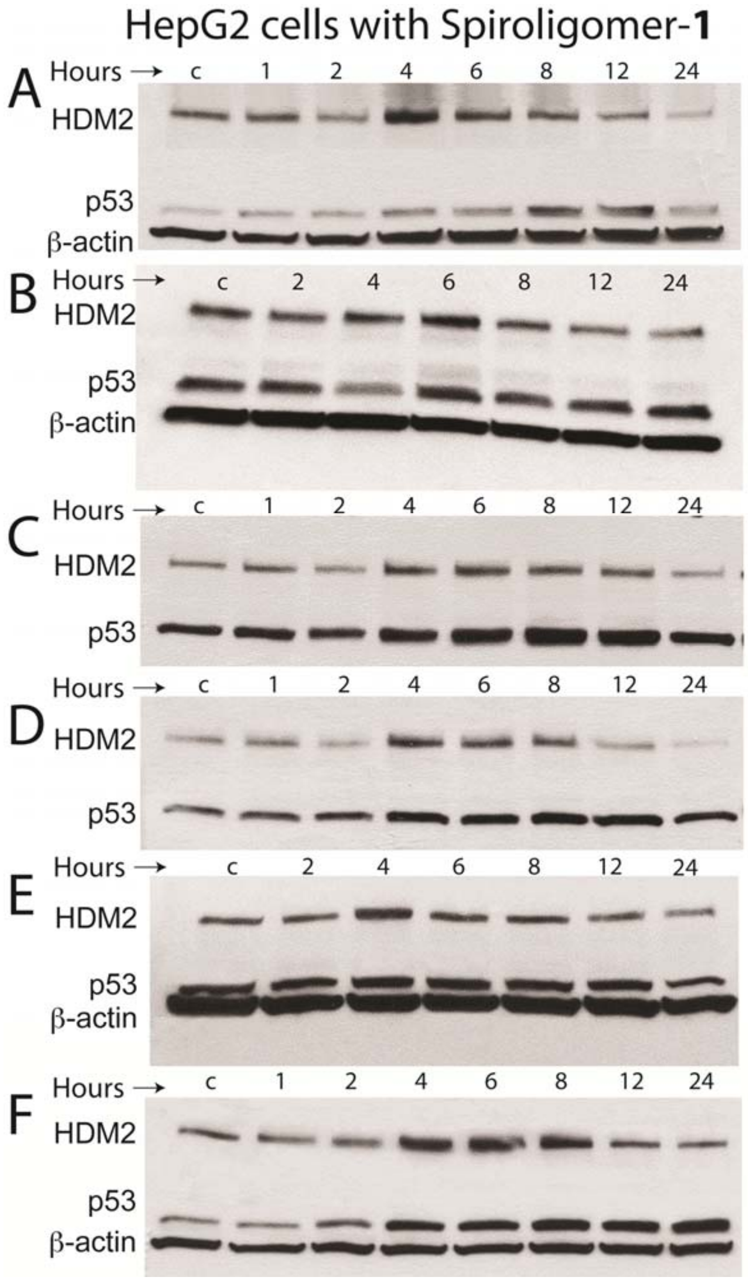

Figure **S.6**. Time-dependent Western blot analysis of spiroligomer **1** in HepG2 cells. Five independent replicates are shown with the levels of HDM2 and p53 as a function of time in hours. All time points were with a concentration of 15 uM of compound **1**.

RNA Extraction, Reverse Transcription and PCR Analysis. Total RNA was extracted using High Pure RNA Isolation Kit (Roche Applied Science) according to enclosed instructions. cDNA was prepared using RETROscript Reverse Transcription Kit (Ambion). *HDM2* primers (Ensembl gene database: HDM2, ENSG00000135679) were 5'-GGTGCTGTAACCACCTCACA-3' (forward) and 5'-TTTTTGTGCACCAACAGACTTT-3' (reverse). These primers recognize cDNAs of all *HDM2* isoforms with p53 binding sequence[7] and generate 102 bp amplicon. β-actin primers for 243 bp PCR transcript (Ensemble gene: Actb, ENSRNOG00000034254) were 5'-TACCACTGGCATTGTGATGG-3' (forward) and 5'-GGGCAACATAGCACAGCTTC-3' (reverse). Primers were designed using Primer3Plus Web Interface. cDNA was amplified using FastStart SYBR Green Master Kit (Roche Applied Science). Real-time quantitative PCR was performed using Stratagene Mx 3005P QPCR system. PCR samples were amplified in 40 cycles; each cycle consisted of 60 sec of denaturation at 95° C, 60 sec of annealing at 60° C, and 60 sec of elongation at 72° C. Mathematical model described[8] and MxPro software (Stratagene) were used to process PCR data. End-point PCR reactions were assayed with PCR Master Mix (Promega). Samples were amplified in 34 cycles; each cycle consisted of 30 sec of denaturation at 95° C, 30 sec of annealing at 60° C, and 30 sec of elongation at 72° C. After 34 cycles, samples were subjected to final elongation for 5 minutes at 72°C. Amplification products were separated by 2% agarose gel electrophoresis, and images of DNA bands were visualized by ethidium bromide staining. See Figure **S.6**.

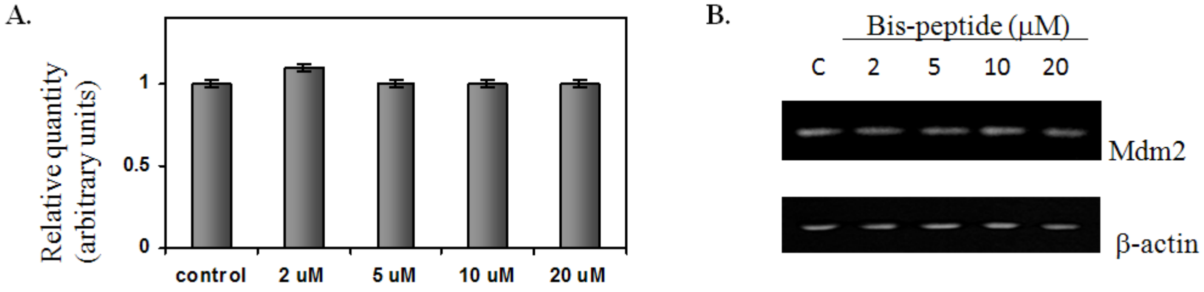

Figure **S.7**. Real-time quantitative PCR (A.) and end-point PCR (B.) analysis of *HDM2* in Huh7 cells treated with indicated concentrations of compound **1** showing no difference in *HDM2* expression levels. The expression of β-actin was used as loading control. Each PCR sample was run in triplicate.

Active vs. Passive Transport Experiments. To further understand the cell-penetration ability of compound **1**, we undertook experiments to provide evidence if an active or passive transport mechanism was operating. HepG2 cells were plated to a density of 1.2 x 10^{5} cells per chamber using the media described above (treatment used 0.5% FBS). Control experiments were

performed by incubating the compound **1** (5 μM concentration) at 37°C as well as fluorescein only (5 μM concentration at 37° C) with HepG2 cells for 3.75 hours. For the 4° C experiments, cells were pretreated at 4° C for 30 min, and then compound **1** was added to the same medium (final concentration of 5 μM). Incubation time at +4° C was 3.75 hours, and ~5% cells became detached after this period of time. For experiments with NaN_3 and deoxy-D-glucose, cells were pretreated with NaN_3 (10 mM) and deoxy-D-glucose (50 mM) for 60 min at +37° C, followed by the addition of compound **1** to the medium (final concentration of 5 μM). Incubation time at +37° C was 3.75 hours, and ~30-40% cells became detached after this period of time.

For all experiments, the media was removed from the chamber after the required incubation time. PBS was then added, and pictures of live cells were taken in PBS. PBS was then removed, cells were fixed (as described above), covered with mounting medium with DAPI, and pictures of fixed cells were taken.

Control experiments, where cells were incubated with fluorescein only, failed to produce any fluorescence in the images.

Figure **S.8**.
Compound **1**
^{1}H NMR in
DMSO at 5 mM

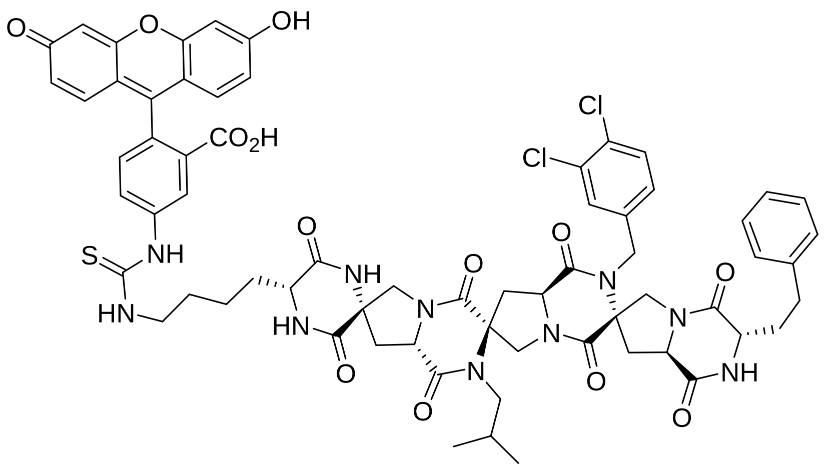

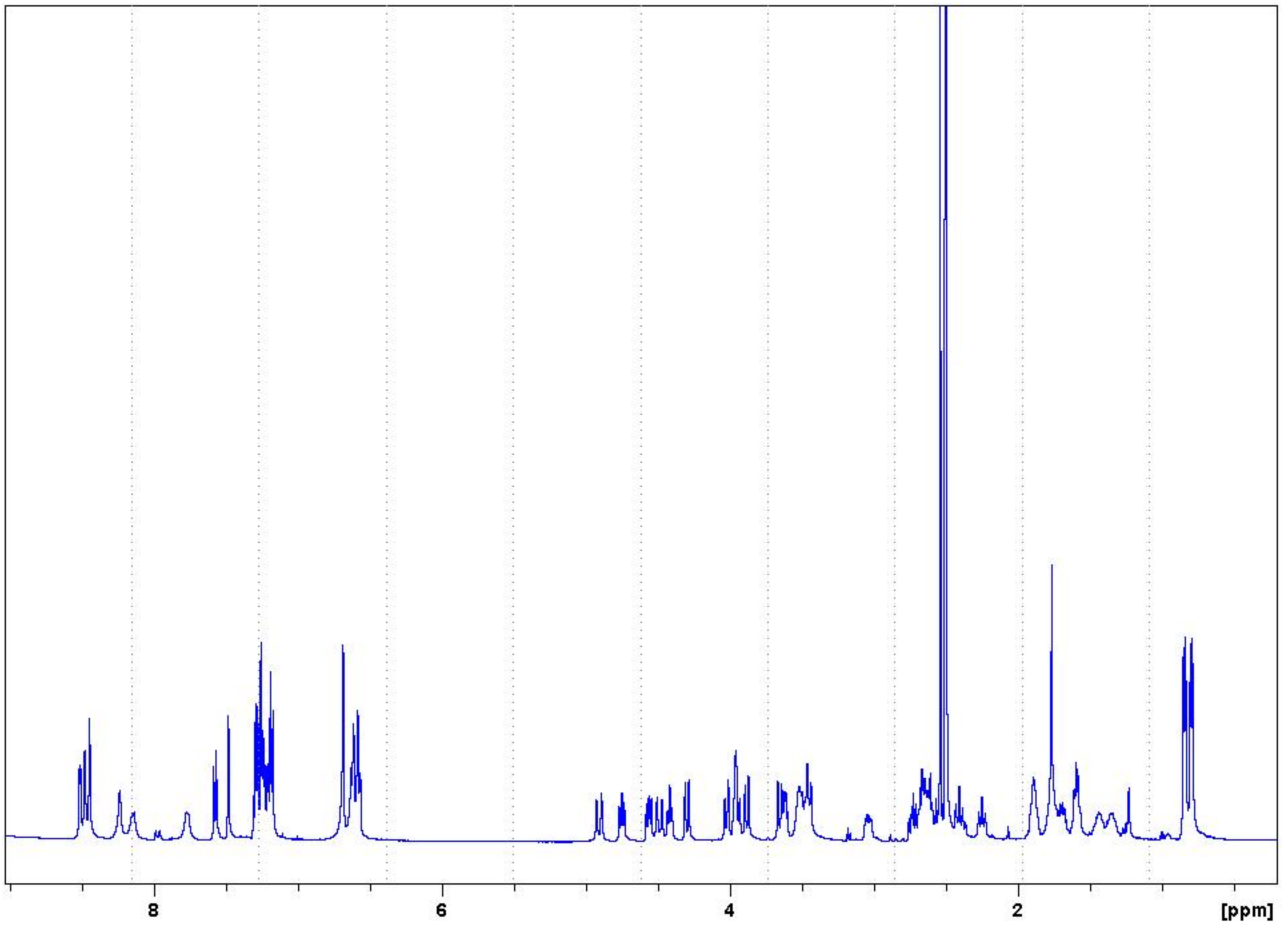

Figure **S.9**.
Compound **1**
HMQC in DMSO
at 5 mM

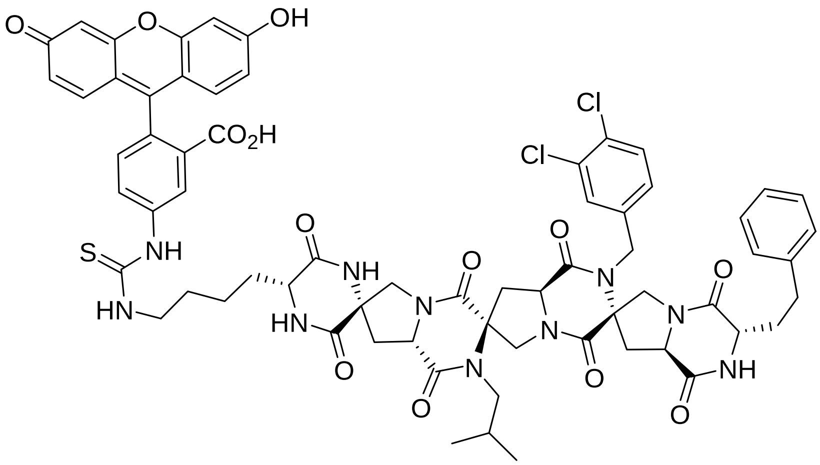

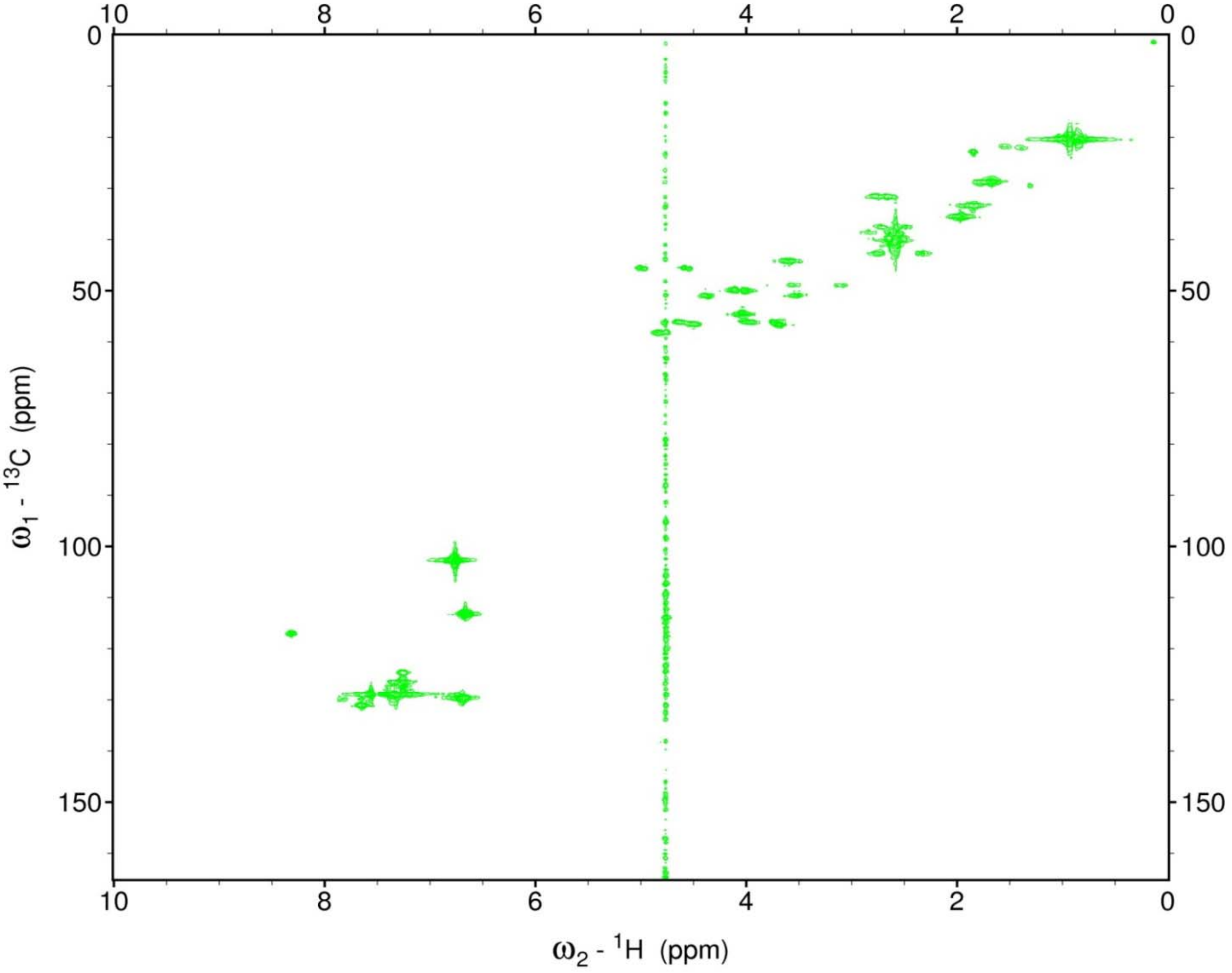

Figure **S.10**.
Compound **1**
HMBC in DMSO
at 5 mM

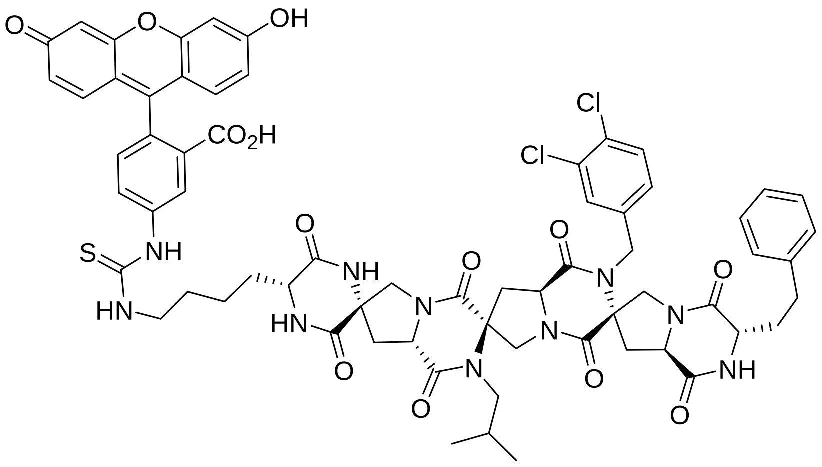

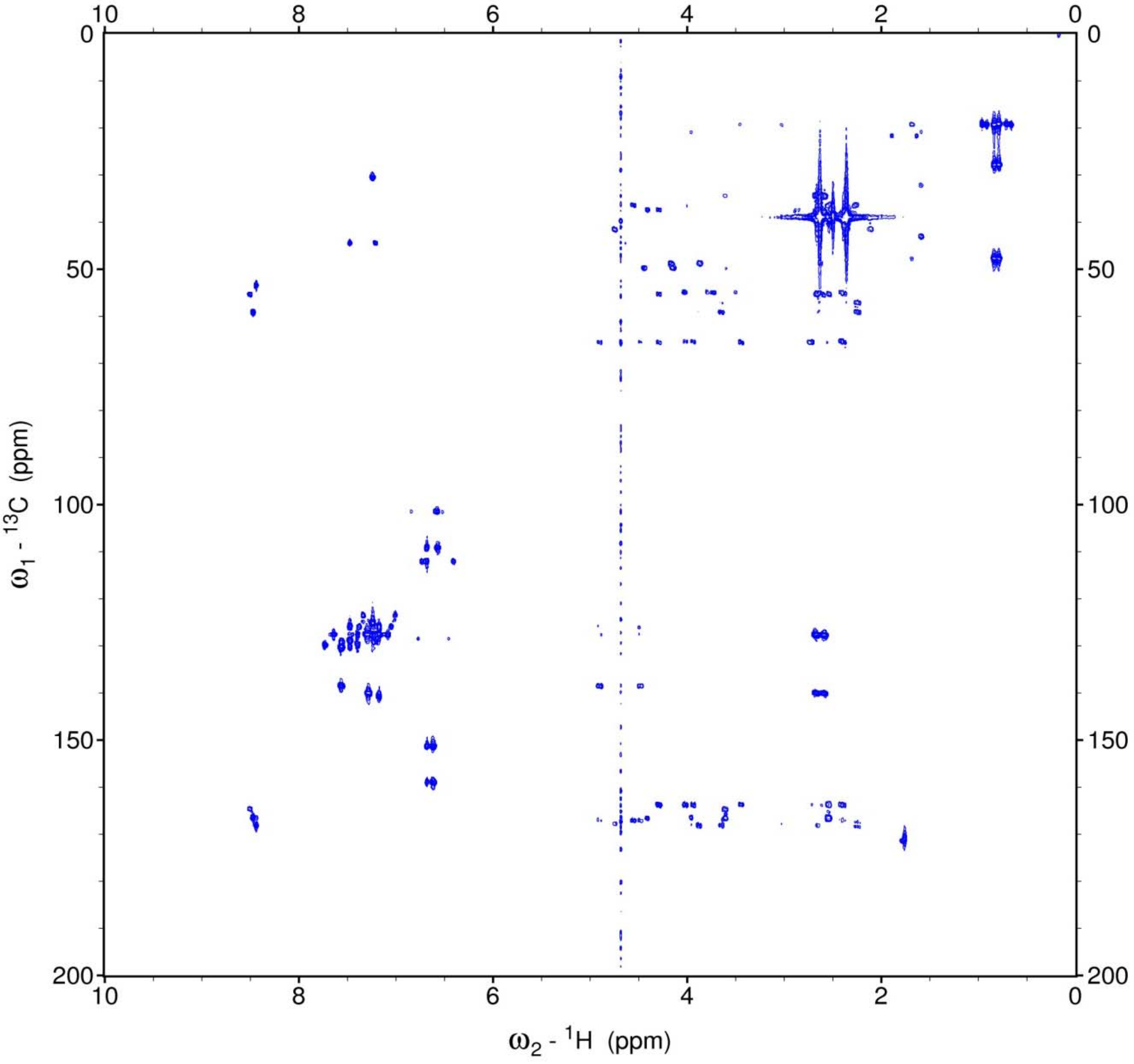

Supporting Information References

1.) Brown, Z. Z. and Schafmeister, C. E. *Org. Lett*. **2010**, *12* (7), 1436

2.) Brown, Z. Z., Alleva, J. and Schafmeister, C. E. *Biopolymers*, **2011***, 96,* 578

3.) Schafmeister, C. E., Brown, Z. Z., and Gupta, S. *Acc. Chem. Res*., **2008**, *41*(10), 1387

4.) Zondlo, S., Lee, A. and Zondlo, N *Biochemistry* **2006** *45*, 11945

5.) Czarna, A., Popowicz, G., Pecak, A., Wolf, S., Dubin G. and Holak T. *Cell Cycle* **2009**, *8*, 1176

6.) http://www.graphpad.com/Prism/organize.htm

7.) Toledo F, Wahl G. M. *Int J Biochem Cell Biol* **2007,** 39, 1476

8.) Pfaffl MW. *Nucl Acids Res* **2001,** 29(9), 2002
